# Antioxidant, Antibacterial, and Antivirulence Activities of a Bioactive Fraction from *Lycopus lucidus* Against *Porphyromonas gingivalis*

**DOI:** 10.3390/antiox15091121

**Published:** 2026-09-04

**Authors:** Jung Min Park, Sohae Park, Dae Youn Hwang, Heeseob Lee, Jumin Park

**Affiliations:** 1 Department of Food Science and Nutrition, College of Human Ecology, Pusan National University, Busan 46241, Republic of Korea; a2022683@pusan.ac.kr (J.M.P.); heeseoblee@pusan.ac.kr (H.L.); 2 Department of Biomaterials Science (BK21 FOUR Program), Life and Industry Convergence Research Institute, College of Natural Resources and Life Science, Pusan National University, Miryang 50463, Republic of Korea; sohaehw@pusan.ac.kr (S.P.); dyhwang@pusan.ac.kr (D.Y.H.)

**Keywords:** *Lycopus lucidus*, Porphyromonas gingivalis, antioxidant activity, antibiofilm activity, antivirulence activity, α-cyperone, gingipain, periodontal pathogen

## Abstract

*Porphyromonas gingivalis* (Pg) is a keystone periodontal pathogen associated with biofilm formation and gingipain-mediated virulence. This study evaluated the antioxidant, antibacterial, antibiofilm, and antivirulence activities of a hexane extract of *Lycopus lucidus* (LLH) and its eight chromatographic fractions (H1–H8). Antioxidant activity was assessed using DPPH and ABTS radical-scavenging assays, whereas antibacterial activity, biofilm formation, and virulence-associated gene expression were evaluated using corresponding in vitro assays. LLH exhibited antioxidant and antibacterial activities, while H4 showed the strongest overall biological activity among the fractions. The DPPH IC_50_ values of LLH and H4 were 98.33 ± 2.05 and 60.67 ± 3.09 µg/mL, respectively, and the corresponding ABTS IC_50_ values were 89.24 ± 1.67 and 28.15 ± 1.21 µg/mL, respectively. H4 also showed greater inhibition of biofilm formation than LLH and more pronounced downregulation of several virulence-associated genes. LC–MS/MS analysis tentatively identified α-cyperone as a constituent of H4. Overall, chromatographic fractionation of LLH yielded H4 with enhanced biological activity across several measured endpoints, including radical-scavenging, antibacterial, and antibiofilm effects, together with more pronounced suppression of several virulence-associated genes. However, the contribution of α-cyperone or other individual constituents to these effects remains to be established.

## 1. Introduction

Periodontitis is a highly prevalent chronic inflammatory disease of the tooth-supporting tissues and represents a significant global health burden [1]. It is driven by dysbiotic plaque biofilms that promote progressive periodontal tissue destruction and tooth loss [2]. In addition to microbial dysbiosis, oxidative stress is an important contributor to periodontal tissue damage. Excessive reactive oxygen species (ROS) generated during host–microbe interactions can amplify inflammatory responses and accelerate periodontal tissue destruction [3,4].

The oral cavity harbors a complex microbial community that influences the development and progression of periodontitis [5,6]. Within this community, *Porphyromonas gingivalis* (Pg), a Gram-negative anaerobe, functions as a keystone pathogen by disrupting host immune homeostasis and promoting microbial dysbiosis and inflammatory bone loss [7,8]. Pg expresses several virulence factors, including lipopolysaccharide, fimbriae, and gingipains, which contribute to bacterial adhesion, biofilm formation, immune evasion, and periodontal tissue damage [9]. Fimbriae facilitate bacterial interactions with host tissues, whereas the arginine-specific and lysine-specific gingipains RgpA and Kgp, respectively, promote extracellular proteolysis, immune dysregulation, and biofilm maturation [10,11,12]. These findings support targeting bacterial virulence and biofilm formation as an alternative or complementary approach to direct bacterial eradication.

Current treatments for periodontitis, including mechanical debridement and adjunctive antimicrobial therapy, have limitations related to biofilm tolerance, incomplete pathogen elimination, and disease recurrence [13,14]. These limitations have increased interest in natural bioactive compounds that can exert antioxidant effects while also interfering with bacterial growth, biofilm formation, and virulence.

*Lycopus lucidus* (LL), a perennial herb of the family Lamiaceae, has been used in traditional medicine for various therapeutic purposes [15]. *Lycopus* species contain diverse bioactive compounds, including flavonoids, coumarins, terpenoids, and tannins, with reported antioxidant, anti-inflammatory, and antimicrobial properties [16]. Previous studies have demonstrated antimicrobial activity of LL extracts against various bacteria and fungi [17]. These findings suggest that LL may represent a natural source of bioactive compounds with antimicrobial potential. Among the solvent extracts evaluated in preliminary screening, the hexane extract (LLH) exhibited the strongest antibacterial activity against Pg and was therefore selected for further chromatographic fractionation. Although the antioxidant and antimicrobial properties of LL extracts have been reported, the biological activities of individual LLH-derived fractions against Pg, particularly their antibacterial, antibiofilm, and antivirulence effects, remain insufficiently characterized. The rise in antibiotic resistance and the limitations of current treatments underscore the need to explore natural sources such as LL for bioactive candidates relevant to periodontitis [9,18].

Therefore, this study evaluated the antioxidant activity of LLH and its chromatographic subfractions and investigated the effects of the LLH subfractions on Pg growth and pathogenic traits, including biofilm formation and virulence-associated gene expression. The chemical constituents of the most active subfraction were further characterized by LC–MS/MS.

## 2. Materials and Methods

### 2.1. Plant Material and Preparation of Extracts

Dried LL leaves were purchased from Dongui Herb Co. (Gwangju, Republic of Korea). A voucher specimen (WPC-23-007) was retained at the Functional Materials Bank (FMB) of the Pusan National University (PNU)–Wellbeing RIS Center. The dried plant material (15 g) was extracted with 300 mL of 80% methanol (MeOH; Daejung, Siheung, Republic of Korea) for 12 h at 25 °C. The initial 80% MeOH extraction was performed to obtain a crude extract containing constituents with a relatively broad range of polarities. After filtration, the solvent was removed under reduced pressure to obtain the crude MeOH extract. The dried crude extract was subsequently extracted with hexane (Junsei Chemical Co., Tokyo, Japan) to recover the relatively nonpolar fraction. Specifically, the dried extract was extracted with hexane (300 mL) for 1 h, and this procedure was repeated three times. The hexane extracts were combined, concentrated to dryness, and designated as the hexane extract (LLH), yielding 6.15 ± 0.19% relative to the 80% MeOH extract. Among the solvent extracts evaluated in preliminary screening, LLH exhibited the strongest antibacterial activity against Pg and was therefore selected for further chromatographic fractionation.

### 2.2. Fractionation by Preparative High-Performance Liquid Chromatography (HPLC)

LLH was fractionated using a preparative HPLC system (LC-forte/R, YMC Co., Kyoto, Japan) equipped with a Triart Prep C18-S column (250 mm × 10.0 mm, 10 µm; YMC Co., Kyoto, Japan). The extract was dissolved in methanol (100 mg/mL) and filtered through a 0.45 µm syringe filter (LBscience, Busan, Republic of Korea). A 400 µL aliquot was injected for each run. The mobile phase consisted of 0.1% formic acid in water (A) and acetonitrile (B), both obtained from Thermo Fisher Scientific Inc. (Waltham, MA, USA). Gradient elution was performed from 55% to 95% B over 25 min, followed by a return to 55% B within 0.1 min and a 6.9 min hold at 55% B, resulting in a total run time of 32 min, at a flow rate of 4.0 mL/min. Fractions were collected based on peak separation monitored by UV absorbance at 210 nm and designated H1–H8 according to their order of elution. The approximate collection windows were 1.5–9.5 min for H1, 9.5–13.0 min for H2, 13.0–16.0 min for H3, 16.0–18.5 min for H4, 18.5–22.5 min for H5, 22.5–25.0 min for H6, 25.0–27.5 min for H7, and 27.5–31.0 min for H8. Eluates corresponding to the same collection window from repeated preparative HPLC runs were pooled and concentrated to yield the individual fractions. The yields of H1–H8, calculated relative to the amount of LLH subjected to preparative HPLC, were 0.541, 1.986, 0.708, 0.777, 0.652, 3.708, 3.291, and 1.416%, respectively.

### 2.3. Liquid Chromatography–Tandem Mass Spectrometry (LC-MS/MS) Analysis

LC–MS/MS analysis was performed using an Agilent 1290 Infinity ultra-high-performance liquid chromatography (UHPLC) system coupled to a hybrid quadrupole time-of-flight (Q-TOF) mass spectrometer (Agilent Technologies, Santa Clara, CA, USA). Chromatographic separation was achieved using a high-strength silica (HSS) T3 column (2.1 mm × 100 mm, 1.8 µm; Waters, Milford, MA, USA) with water (A) and acetonitrile (B) as the mobile phases at a flow rate of 0.4 mL/min. A 10 µL aliquot was injected for each analysis. The gradient program was as follows: 5% B at 1 min, increased to 100% B by 17 min and held until 18 min, returned to 5% B at 18.1 min, and held at 5% B until 20 min. Mass spectrometric detection was performed in electrospray ionization mode. The source parameters were as follows: gas temperature, 300 °C; gas flow, 9 L/min; nebulizer pressure, 45 psig; sheath gas temperature, 350 °C; sheath gas flow, 11 L/min; VCap, 4000 V; and fragmentor voltage, 175 V. MS/MS experiments were performed with collision energies of 10–20 V. The LC–MS/MS conditions used in this study were specifically established for the chemical profiling of LLH and H4. Tentative compound identification was based on accurate mass measurements, chromatographic retention behavior, and MS/MS fragmentation patterns. The acquired MS/MS spectra of LLH and H4 were compared with reference spectra in the NIST spectral library using the library-matching function of Agilent MassHunter Qualitative Analysis software (version 12.0; Agilent Technologies, Santa Clara, CA, USA). Where available, MassBank entries and previously reported spectral data were also consulted to support the tentative assignments. Because authentic standards and complementary structural techniques such as NMR were not used, these assignments should be regarded as tentative rather than definitive. For compounds with possible positional or geometric isomers, the exact positions of unsaturation or substitution and stereochemical configurations were not independently confirmed in this study.

### 2.4. Antioxidant Assays

#### 2.4.1. DPPH Radical Scavenging Assay

DPPH radical-scavenging activity was measured using a modified DPPH assay [19]. A 60 μM solution of 2,2-diphenyl-1-picrylhydrazyl (DPPH; Sigma-Aldrich, St. Louis, MO, USA) was prepared in 95% ethanol (Junsei Chemical Co., Tokyo, Japan) and filtered before use. The DPPH solution (100 μL) was mixed with an equal volume of each sample solution to obtain final sample concentrations ranging from 8 to 500 μg/mL. The mixtures were incubated in the dark at room temperature for 30 min, and the absorbance was measured at 520 nm using a microplate reader (Sunrise; Tecan, Graz, Austria). Ascorbic acid (Sigma-Aldrich) was used as a positive control and evaluated under the same assay conditions. The concentration required to scavenge 50% of DPPH radicals (IC_50_) was determined from the concentration–response curve.

#### 2.4.2. ABTS Radical Scavenging Assay

ABTS radical-scavenging activity was measured using the ABTS decolorization assay [20]. A 7 mM solution of 2,2′-azino-bis(3-ethylbenzothiazoline-6-sulfonic acid) (ABTS) and a 2.45 mM potassium persulfate solution (both fromSigma-Aldrich, St. Louis, MO, USA) were mixed at a ratio of 2:1 and incubated in the dark at room temperature for 12 h. The resulting solution was diluted with 5 mM phosphate-buffered saline (PBS; pH 7.4) to an absorbance of 0.70 ± 0.02 at 734 nm. Each sample solution (10 μL) was mixed with 190 μL of the ABTS working solution to obtain final sample concentrations ranging from 8 to 500 μg/mL. The mixtures were incubated in the dark at room temperature for 6 min, and the absorbance was measured at 734 nm using a spectrophotometer (Thermo Fisher Scientific, Waltham, MA, USA). Trolox (Sigma-Aldrich) was used as a positive control and evaluated under the same assay conditions. ABTS radical-scavenging activity was expressed as percentage inhibition relative to the control. The concentration required to scavenge 50% of ABTS radicals (IC_50_) was determined from the concentration–response curve.

#### 2.4.3. Total Phenolic Content

Total phenolic content (TPC) was measured using a modified Folin–Ciocalteu method [21]. Each sample solution (50 μL) was mixed with 500 μL of distilled water and 100 μL of Folin–Ciocalteu reagent (Sigma-Aldrich, St. Louis, MO, USA). After incubation in the dark for 3 min, 100 μL of 10% sodium nitrate solution (Sigma-Aldrich) and 350 μL of distilled water were added to each well. The mixtures were incubated in the dark at 25 °C for 1 h, and the absorbance was measured at 725 nm. TPC was determined using a caffeic acid (Sigma-Aldrich) standard curve over a concentration range of 50–450 µg/mL, with a coefficient of determination (R^2^) of 0.9964. The results were expressed as milligrams of caffeic acid equivalents per gram of dry matter of the extract (mg CAE/g DM).

#### 2.4.4. Total Flavonoid Content

Total flavonoid content (TFC) was measured using a modified diethylene glycol colorimetric method [22]. Each sample solution (100 μL) was mixed with 1000 μL of 90% diethylene glycol solution (Daejung, Siheung, Republic of Korea). Sodium hydroxide solution (100 μL; Duksan Pure Chemicals Co., Ltd., Ansan, Republic of Korea) was added, and the mixture was incubated at 37 °C for 1 h. The absorbance was measured at 415 nm using a microplate reader (Sunrise; Tecan). TFC was determined using a naringin (Sigma-Aldrich) standard curve over a concentration range of 50–450 µg/mL, with a coefficient of determination (R^2^) of 0.9965. The results were expressed as milligrams of naringin equivalents per gram of dry matter of the extract (mg NE/g DM).

### 2.5. Antimicrobial Assay

#### 2.5.1. Bacterial Strain

Pg (KCTC 5352) was obtained from the Korean Collection for Type Cultures (KCTC, Daejeon, Republic of Korea). The strain was cultured anaerobically at 37 °C for 24 h in tryptic soy broth (TSB; Difco Laboratories, Detroit, MI, USA) supplemented with 0.5% yeast extract (Difco Laboratories), 5 µg/mL hemin (Sigma-Aldrich), and 1 µg/mL menadione (Sigma-Aldrich), under conditions modified from those previously described [23]. For agar-based assays, TSB agar supplemented with 5% (*v*/*v*) defibrinated sheep blood (KisanBio, Seoul, Republic of Korea) was used. Anaerobic conditions were maintained using an anaerobic jar equipped with a gas-generating system.

#### 2.5.2. Disc Diffusion Assay

The antimicrobial activity of LLH and its fractions against Pg was evaluated using the disc diffusion method, with modifications to a previously described protocol [24]. Bacterial suspensions were adjusted to an optical density of 0.1 at 600 nm and uniformly spread onto TSB agar plates supplemented with 5% sheep blood. Sterile 8 mm paper discs (ADVANTEC, Tokyo, Japan) were impregnated with 50 µL of each sample solution to achieve the indicated loading amounts (expressed as mg/disc) and placed on the inoculated plates. Discs containing dimethyl sulfoxide (DMSO; Duchefa Biochemie, Haarlem, Netherlands) alone served as negative controls. The plates were incubated anaerobically at 37 °C for 24 h, and the inhibition zones were measured in millimeters.

#### 2.5.3. Determination of the Minimum Inhibitory/Bactericidal Concentration (MIC/MBC)

The MICs of LLH and its fractions against Pg were determined using a broth microdilution assay with modifications to a previously described method [25]. Stock solutions of each sample were prepared in DMSO and further diluted in supplemented TSB to obtain final concentrations ranging from 4 to 1000 µg/mL. The final DMSO concentration did not exceed 1% (*v*/*v*), and solvent control wells containing equivalent DMSO concentrations were included. The bacterial inoculum was standardized as described in Section 2.5.1 and added to each well to achieve a final volume of 200 µL. The plates were incubated anaerobically at 37 °C for 24 h. The MIC was defined as the lowest concentration at which no visible bacterial growth was observed, as confirmed by measuring the absorbance at 600 nm using a microplate reader (Sunrise; Tecan).

For MBC determination, 100 µL aliquots from wells showing no visible growth were spread onto TSB agar plates supplemented with 5% sheep blood and incubated anaerobically at 37 °C for 24 h. The MBC was defined as the lowest concentration at which no colony growth was observed.

#### 2.5.4. Evaluation of Bacterial Growth Curve

Bacterial growth kinetics were evaluated using a modified broth microdilution method [26]. The LLH fractions were dissolved in DMSO and added to bacterial suspensions to achieve the desired final concentrations. A standardized suspension of Pg was dispensed into 96-well plates (SPL Life Sciences Co., Pocheon, Republic of Korea) to a final volume of 200 µL per well. The final DMSO concentration did not exceed 1% (*v*/*v*), and control wells contained bacterial suspensions with an equivalent concentration of DMSO but no test fraction. The plates were incubated anaerobically at 37 °C, and the optical density at 600 nm (OD_600_) was measured every 4 h for 24 h using a microplate reader (Sunrise; Tecan). Growth curves were generated by plotting OD_600_ values against time.

### 2.6. Antibiofilm and Antivirulence Assays

#### 2.6.1. Antibiofilm Assay

The antibiofilm activities of the LLH fractions were evaluated using a modified crystal violet assay [27]. Activated Pg cultures were dispensed into 96-well plates (final volume, 100 µL per well), and LLH fractions were added to achieve final concentrations ranging from 8 to 1000 µg/mL. The final DMSO concentration did not exceed 1% (*v*/*v*), and solvent control wells containing only DMSO were included. The plates were incubated anaerobically at 37 °C for 72 h to allow biofilm formation. Following incubation, the wells were gently washed with phosphate-buffered saline (PBS) to remove non-adherent cells from the wells. Adherent biofilms were fixed with 99% methanol and stained with 0.1% crystal violet (Sigma-Aldrich, St. Louis, MO, USA) for 15 min. Excess stain was removed by washing, and the bound dye was solubilized using 33% acetic acid (Daejung, Siheung, Republic of Korea). Absorbance was measured at 590 nm using a microplate reader (Sunrise; Tecan).

#### 2.6.2. Quantitative Real-Time PCR (qRT-PCR) Analysis of Gene Expression

Gene expression in Pg was analyzed by qRT-PCR. Bacterial cultures were treated with LLH or the H4 fraction for 24 h, and untreated cultures served as controls. Total RNA was extracted using the EZ™ Total RNA Miniprep Kit (Enzynomics, Daejeon, Republic of Korea), and qRT-PCR was performed using the TOPreal™ One-step qRT-PCR Kit (Enzynomics) on a CFX Connect Real-Time PCR System (Bio-Rad Laboratories, Inc., Hercules, CA, USA). The expression levels of the virulence-associated genes *fimA, rgpA, kgp,* and *htrA* were normalized to 16S rRNA and calculated using the 2^−ΔΔCt^ method. The primer sequences are provided in Table S1.

### 2.7. Statistical Analysis

Data are presented as the mean ± standard deviation (SD). Statistical differences were analyzed by one-way ANOVA followed by Duncan’s multiple range test using SPSS (version 25.0). Differences were considered statistically significant at *p* < 0.05.

## 3. Results

### 3.1. Fractionation and LC–MS/MS Profiling of LLH and H4

**Figure 1:**
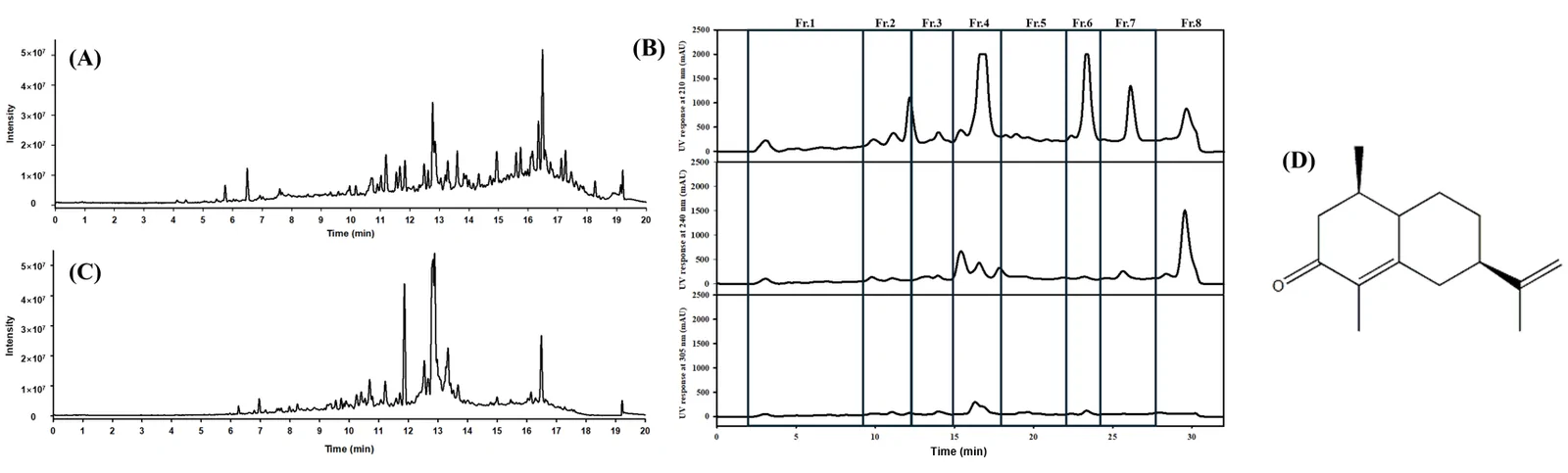
Chemical profiling and fractionation of *Lycopus lucidus* hexane extract (LLH). (**A**) Total ion chromatogram of LLH obtained using UHPLC–ESI–MS in positive ion mode. (**B**) Preparative HPLC chromatograms of LLH monitored at 210, 240, and 305 nm during fractionation. (**C**) Total ion chromatogram of the bioactive H4 fraction analyzed by UHPLC–ESI–MS in positive ion mode. (**D**) Chemical structure of α-cyperone.

LLH was fractionated by preparative HPLC based on its analytical chromatographic profile. The total ion chromatogram (TIC) of LLH obtained by UHPLC–ESI–MS in positive ion mode is shown in Figure 1A. UV chromatograms recorded at 210, 240, and 305 nm revealed multiple distinct peaks, indicating the chemical complexity of the extract (Figure 1B). Preparative separation yielded eight fractions (H1–H8), which were subsequently evaluated for antioxidant and antimicrobial activities. Following biological screening, H4 was selected for further chemical characterization by LC–MS/MS.

Multiple compounds in LLH were tentatively identified based on accurate mass measurements, retention times, and available MS/MS spectral information (Table 1). The detected constituents included terpenoids, fatty acids, and related derivatives. Compared with LLH, H4 exhibited a less complex chromatographic profile with fewer dominant peaks (Figure 1C). Among the detected compounds, α-cyperone was tentatively identified as a constituent of H4 based on its accurate [M+H]^+^ mass (observed *m/z* 219.1739; mass error, −1.83 ppm) and MS/MS fragmentation pattern, which was compared with spectral information available in the NIST spectral library, MassBank database, and relevant literature (Table 2). The proposed chemical structure of α-cyperone is shown in Figure 1D. Because an authentic α-cyperone standard and complementary structural techniques such as NMR were not used, this assignment should be regarded as tentative rather than definitive. Additional compounds, including cinnamic acid derivatives and fatty acids, were also detected in H4. These results indicate differences in the chemical composition of H4 compared with LLH.

### 3.2. Antioxidant Properties

The radical-scavenging activities of LLH and its fractions were evaluated using DPPH and ABTS assays, while total phenolic content (TPC) and total flavonoid content (TFC) were determined as complementary phytochemical indices to provide additional context for the radical-scavenging results (Table 3). Among the samples tested, H4 exhibited the strongest DPPH radical-scavenging activity, as indicated by the lowest IC_50_ value of 60.67 ± 3.09 μg/mL, followed by H1 (78.50 ± 2.68 μg/mL), LLH (98.33 ± 2.05 μg/mL), H2 (116.57 ± 5.43 μg/mL), H8 (218.97 ± 2.72 μg/mL), and H7 (316.67 ± 5.44 μg/mL). The DPPH IC_50_ values of H3, H5, and H6 exceeded 500 μg/mL. Similarly, H4 exhibited the strongest ABTS radical-scavenging activity, with an IC_50_ value of 28.15 ± 1.21 μg/mL, followed by H1 (42.05 ± 2.78 μg/mL), LLH (89.24 ± 1.67 μg/mL), H2 (95.16 ± 2.91 μg/mL), H8 (118.74 ± 2.46 μg/mL), H7 (162.06 ± 1.32 μg/mL), and H3 (325.65 ± 4.22 μg/mL). The ABTS IC_50_ values of H5 and H6 exceeded 500 μg/mL. Ascorbic acid, used as the positive control in the DPPH assay, exhibited an IC_50_ value of 8.25 ± 0.89 μg/mL, whereas Trolox, used as the positive control in the ABTS assay, exhibited an IC_50_ value of 5.12 ± 0.42 μg/mL.

In contrast, H1 exhibited the highest TPC among the fractions, with a value of 253.52 ± 0.67 mg CAE/g DM, whereas LLH exhibited the highest TFC among all samples, with a value of 70.60 ± 3.08 mg NE/g DM. H4 showed intermediate TPC and TFC values of 131.14 ± 1.17 mg CAE/g DM and 34.14 ± 1.47 mg NE/g DM, respectively. Despite these intermediate TPC and TFC values, H4 exhibited the strongest DPPH and ABTS radical-scavenging activities.

### 3.3. Antimicrobial Activities Against Pg

#### 3.3.1. Inhibition Zone

The antimicrobial activities of LLH and its fractions against Pg were evaluated using a disc diffusion assay (Table 4). Inhibitory activity was observed for LLH and all fractions at higher loading amounts, with clear differences in inhibition zone diameters among the samples. At 2 mg/disc, H4 exhibited the largest inhibition zone (23.31 ± 0.16 mm), followed by H3 (21.53 ± 0.09 mm), H2 (20.73 ± 0.09 mm), and LLH (20.34 ± 0.13 mm). In contrast, H6, H7, and H8 showed smaller inhibition zones under the same conditions. No inhibition was observed in the negative control (DMSO).

#### 3.3.2. MIC and MBC

The MIC and MBC values of LLH and its fractions against Pg are presented in Table 4. MIC values ranged from 64 to 500 µg/mL, whereas MBC values ranged from 250 to ≥1000 µg/mL among the tested samples. H4, H3, and LLH exhibited the lowest MIC value of 64 µg/mL. H4 showed an MBC of 250 µg/mL, whereas LLH and H3 exhibited higher MBC values. In contrast, H1 and H5 showed higher MIC and MBC values, indicating comparatively lower antibacterial activity.

#### 3.3.3. Bacterial Growth Curve

**Figure 2:**
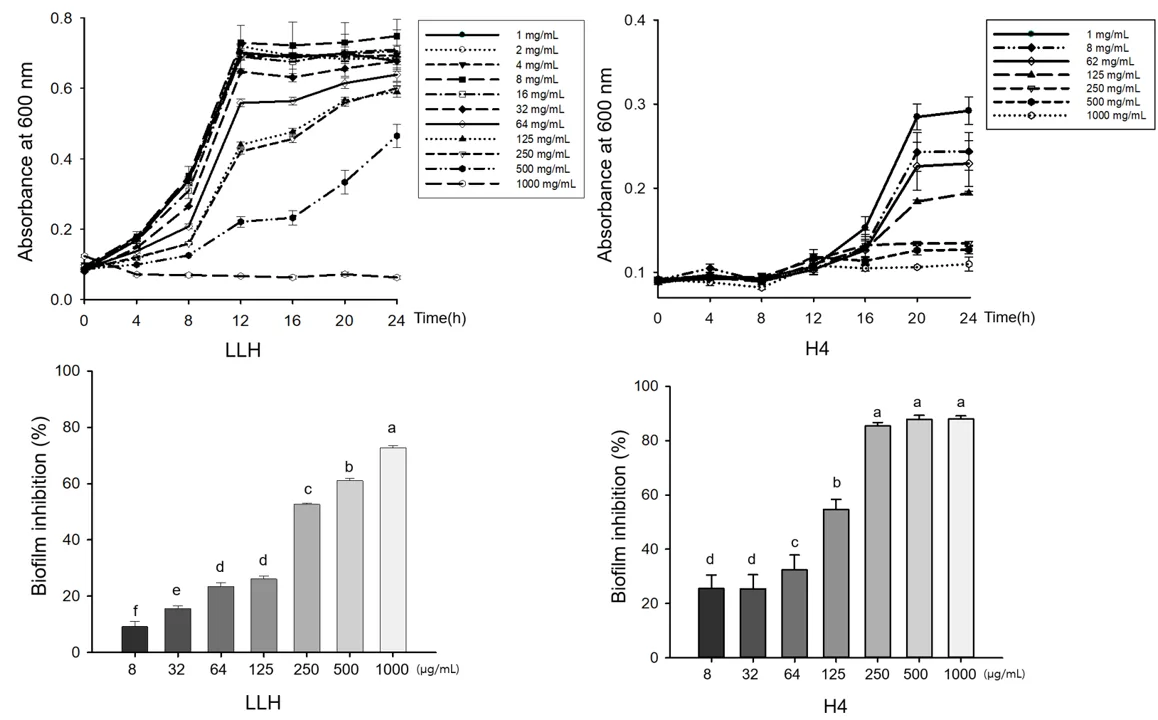
Effects of *Lycopus lucidus* hexane extract (LLH) and H4 fraction on the growth kinetics and biofilm formation of *Porphyromonas gingivalis* (Pg). (**A**) Growth curves of Pg treated with serial concentrations of LLH. (**B**) Growth curves of Pg treated with serial concentrations of H4 fraction. (**C**) Inhibition of biofilm formation by LLH at different concentrations. (**D**) Inhibition of biofilm formation by the H4 fraction at different concentrations. Data are presented as mean ± standard deviation (SD) (*n* = 3). Different lowercase letters within the same group indicate statistically significant differences (*p* < 0.05; one-way ANOVA, followed by Duncan’s multiple range test).

The growth kinetics of Pg treated with LLH and the H4 fraction are shown in Figure 2A,B. In the untreated control, OD_600_ values increased markedly after 8 h and reached a plateau between 12 and 24 h. Treatment with LLH resulted in concentration-dependent inhibition of bacterial growth (Figure 2A). At higher concentrations, bacterial growth was delayed, and OD_600_ values remained lower than those of the control. H4 showed a stronger inhibitory pattern (Figure 2B). Growth suppression became evident after 12 h, and OD_600_ values remained substantially lower than those of the control and the LLH-treated groups at comparable concentrations.

### 3.4. Analysis of the Antibacterial Mechanisms of the LLH Fractions

#### 3.4.1. Antibiofilm Activity

As shown in Figure 2C,D, both LLH and H4 significantly inhibited biofilm formation by Pg in a concentration-dependent manner (*p* < 0.05). LLH inhibited biofilm formation by approximately 57% at 250 µg/mL and by more than 70% at 1000 µg/mL. In comparison, H4 showed greater inhibitory activity, with biofilm inhibition exceeding 80% at 250 µg/mL and remaining high at higher concentrations. Significant differences between LLH and H4 were observed at equivalent concentrations of ≥125 µg/mL (*p* < 0.05). These results indicate that H4 exhibited greater antibiofilm activity than LLH under the tested conditions.

#### 3.4.2. Effects on Virulence-Related Gene Expression

**Figure 3:**
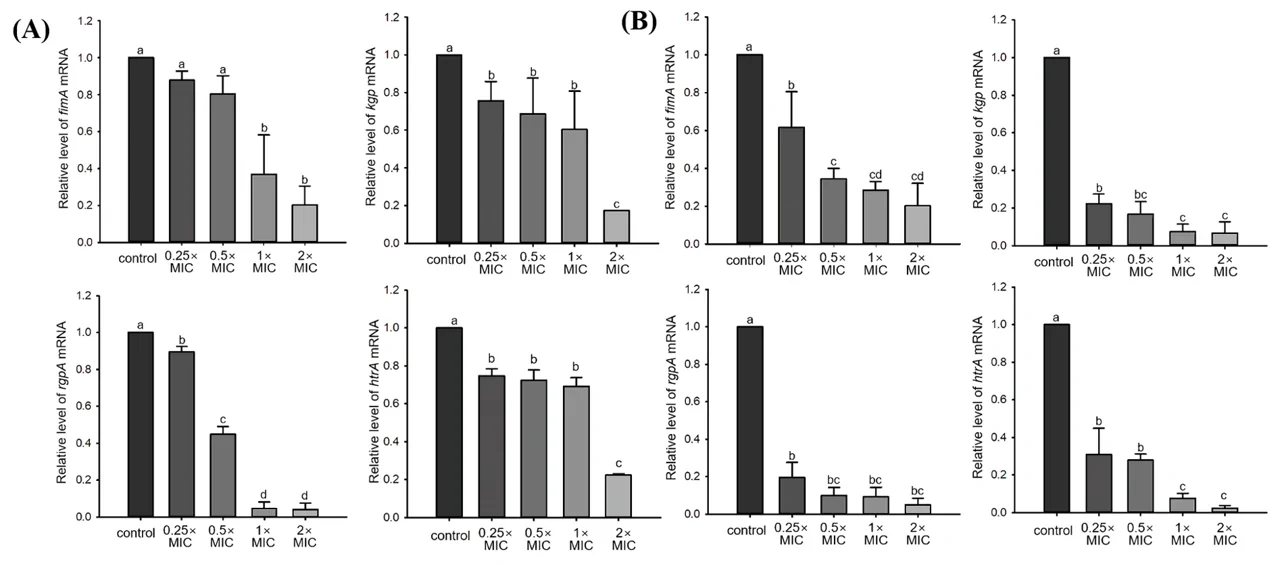
Effects of *Lycopus lucidus* hexane extract (LLH) and the H4 fraction on virulence-related gene expression in *Porphyromonas gingivalis*. (**A**) Relative mRNA expression levels of fimbrillin (*fimA*), arginine-specific gingipain (*rgpA*), lysine-specific gingipain (*kgp*), and high-temperature requirement protein A (*htrA*) following treatment with LLH. (**B**) Relative mRNA expression levels of *fimA, rgpA, kgp,* and *htrA* following treatment with the H4 fraction. Gene expression levels were normalized to 16S rRNA. Data are presented as mean ± standard deviation (SD) (*n* = 3). Different lowercase letters indicate statistically significant differences (*p* < 0.05; one-way ANOVA followed by Duncan’s multiple range test).

The effects of LLH and H4 on the expression of virulence-associated genes in Pg were evaluated by qRT-PCR (Figure 3A,B). The relative expression levels of *fimA*, *rgpA*, *kgp*, and *htrA* were normalized to 16S rRNA. As shown in Figure 3A, LLH treatment significantly reduced the expression of all tested genes compared with the untreated control (*p* < 0.05). A concentration-dependent decrease was particularly evident for *rgpA*, *kgp*, and *htrA*, whereas the reduction in *fimA* expression was less pronounced across the tested concentrations. As shown in Figure 3B, H4 produced more pronounced reductions in the expression of several virulence-associated genes. Downregulation was observed at 0.25× MIC, and the expression levels generally decreased with increasing concentrations. The expression of *rgpA*, *kgp*, and *htrA* was more strongly reduced by H4 than by LLH at equivalent MIC-based concentrations.

## 4. Discussion

Chromatographic fractionation of LLH produced fractions with distinct antioxidant profiles. Although hexane preferentially extracts relatively non-polar constituents and is therefore not generally expected to yield phenolic- or flavonoid-rich fractions, TPC and TFC were included as complementary indices to examine whether the observed radical-scavenging activities of LLH and its fractions were associated with their phenolic and flavonoid contents. Among the H1–H8 fractions, H4 exhibited the strongest radical-scavenging activity, as indicated by the lowest DPPH and ABTS IC_50_ values, despite showing intermediate TPC and TFC values. This lack of direct correspondence suggests that the strong radical-scavenging activity of H4 cannot be explained solely by its total phenolic or flavonoid content and may reflect differences in its overall chemical composition. Previous studies on phenolic-rich polar extracts have reported strong positive correlations between total phenolic content and antioxidant activity [28,29]. In contrast, the present study focused on a hexane-derived extract and its chromatographic fractions, in which relatively non-polar constituents may contribute to the observed antioxidant profile. LC–MS/MS profiling tentatively identified terpenoids and fatty acids in LLH and H4, with α-cyperone among the tentatively identified constituents of H4. However, because quantitative concentrations of the individual constituents were not determined, formal correlations between specific compounds and DPPH or ABTS radical-scavenging activity could not be established. Therefore, the antioxidant activity of H4 cannot be attributed to α-cyperone or any other specific constituent based on the present data.

Previous studies have also demonstrated the considerable antioxidant potential of LL. Rosmarinic acid and luteolin glycosides have been identified as major contributors to its antioxidant activity [16]. Five compounds isolated from the aerial parts of LL—rosmarinic acid, methyl rosmarinate, ethyl rosmarinate, luteolin, and luteolin-7-O-β-D-glucuronide methyl ester—exhibited potent superoxide-scavenging activities, with ethyl rosmarinate showing the lowest IC_50_ value among the isolated compounds [30]. In a previous solvent-fractionation study, the ethyl acetate fraction exhibited particularly strong antioxidant activity, which was attributed to its high rosmarinic acid content and the contribution of luteolin glycosides [16]. In addition, water extracts prepared from the leaves showed higher total polyphenol and flavonoid contents and stronger radical-scavenging activities than those prepared from the stems and roots [31]. Collectively, these findings indicate that the antioxidant capacity of LL is influenced by the distribution and composition of its active constituents, which may vary according to the plant part, extraction solvent, and fractionation procedure. This is consistent with the present finding that H4 exhibited the strongest radical-scavenging activity despite not having the highest TPC or TFC.

Beyond its antioxidant activity, H4 showed antibacterial, antibiofilm, and antivirulence effects against Pg, including inhibition of growth, biofilm formation, and virulence-associated gene expression. The reduced expression of *rgpA*, *kgp*, *fimA*, and *htrA* indicates that H4 affected multiple virulence-associated genes at the transcriptional level. However, the precise regulatory mechanisms underlying these transcriptional changes remain to be determined. Direct comparison with the parent LLH extract showed that fractionation enhanced several measured activities. H4 produced stronger growth inhibition than LLH and showed greater inhibition of biofilm formation at equivalent concentrations. H4 also produced more pronounced downregulation of several virulence-associated genes than LLH at equivalent MIC-based concentrations. These findings indicate that chromatographic fractionation yielded H4 with enhanced activity across several measured endpoints compared with the parent LLH extract. However, the present data do not identify which individual constituent or combination of constituents is responsible for these differences.

Oxidative stress is closely involved in the pathogenesis of periodontitis, in which excessive ROS generated during host–microbe interactions can amplify inflammatory responses and contribute to periodontal tissue damage [32,33,34]. Previous studies using Pg-related cellular models have also shown that antioxidant compounds can reduce ROS production and the secretion of inflammatory mediators, providing a biological rationale for examining antioxidant activity together with antibacterial and antiviral effects [35]. However, the antioxidant effects observed in the present study were demonstrated using chemical radical-scavenging assays, and cellular ROS production, inflammatory responses, and host-protective effects were not directly evaluated. Therefore, the present findings do not establish a causal relationship between the antioxidant activity of H4 and its anti-Pg effects.

Pg is an important periodontal pathogen associated with periodontitis [36,37] and is widely regarded as a keystone pathogen capable of disrupting host–microbiome homeostasis through immune modulation and promotion of dysbiotic microbial communities [38,39]. Central to its pathogenicity are gingipains (RgpA and Kgp), which function as extracellular proteases and regulators of nutrient acquisition, biofilm architecture, and immune evasion [40,41,42]. Gingipain activity contributes to complement degradation, cytokine manipulation, and disruption of epithelial barrier integrity [43,44,45]. Therefore, the reduced expression of *rgpA* and *kgp* observed following H4 treatment indicates transcriptional effects on gingipain-associated virulence genes. However, gingipain enzymatic activity was not directly measured, and the functional consequences of these transcriptional changes remain to be established.

The concurrent downregulation of *htrA* was notable. *HtrA* proteases are implicated in stress adaptation, protein quality control, and bacterial survival under adverse environmental conditions [46,47,48]. H4 treatment reduced *htrA* expression; however, the present study did not evaluate whether this transcriptional change altered the bacterial stress tolerance or fitness.

H4 also inhibited biofilm formation and reduced the expression of several biofilm- and virulence-associated genes. Biofilm development in Pg involves fimbrial adhesion, coaggregation, and gingipain-associated processes [49,50,51]. In the present study, H4 treatment reduced *fimA* expression together with *rgpA* and *kgp* expression and inhibited biofilm formation. These findings are consistent with the effect of H4 on biofilm-associated processes. However, adhesion, coaggregation, matrix remodeling, and other specific mechanisms underlying the observed biofilm inhibition were not directly evaluated.

In the present study, H4 treatment was associated with reduced expression of multiple virulence-associated genes; however, the regulatory pathways responsible for these transcriptional changes were not investigated. Therefore, the observed gene expression changes should be interpreted as an experimentally demonstrated response to H4 treatment rather than evidence of a specific regulatory mechanism.

Importantly, targeting virulence regulation represents a strategic shift from conventional bactericidal approaches that directly inhibit bacterial growth or survival. Antivirulence strategies aim to attenuate pathogenic capacity rather than directly eliminate bacterial cells and have therefore been proposed to impose lower selective pressure for the development of resistance than conventional bactericidal approaches [52]. However, because H4 exhibited both antibacterial and antivirulence activities in the present study, a lower selective pressure cannot be assumed for H4 itself. Furthermore, reduced selective pressure does not imply an absence of resistance risk. Bacterial adaptation and resistance to antivirulence interventions have been experimentally demonstrated in specific antivirulence systems [53], indicating that the possibility of resistance development cannot be completely excluded. In chronic biofilm-associated infections, such as periodontitis, where complete eradication of microbial communities is neither feasible nor desirable, modulation of keystone pathogen virulence may represent a complementary approach to conventional antimicrobial strategies [54,55].

α-Cyperone was tentatively identified as a constituent of H4; however, its individual biological activity was not evaluated in this study. Therefore, the enhanced activity of H4 cannot be attributed specifically to α-cyperone. Other constituents detected in H4 may have also contributed to the observed effects. Further studies are needed to determine the individual contributions of the constituents detected in H4.

Several limitations should be considered. First, virulence suppression was assessed at the transcriptional level, and corresponding reductions in gingipain enzymatic activity were not directly measured. Second, the regulatory pathways affected by H4 have not been characterized, and its impact on global transcriptomic profiles remains unknown. Third, antibiofilm activity was evaluated using a single-species Pg model, and its effects on multispecies oral biofilms remain to be determined. Fourth, the effects of LLH-derived compounds on host epithelial or immune responses, including cellular oxidative and inflammatory responses, were not evaluated. Finally, although α-cyperone was tentatively identified as a constituent of H4, the present findings do not establish that it is responsible for the biological activities of H4, and contributions from other constituents or interactions among them cannot be excluded. Future studies integrating gingipain activity assays, molecular profiling, multispecies biofilm models, and host-cell-based analyses will be necessary to further elucidate the biological mechanisms and relevance of H4.

In summary, H4 inhibited Pg growth, biofilm formation, and virulence-associated gene expression. These findings support further investigation of LLH-derived constituents to determine their individual contributions to the observed in vitro effects.

## 5. Conclusions

This study demonstrated the in vitro radical-scavenging activity of LLH and its chromatographic fractions, as well as their antibacterial and antibiofilm activities and effects on virulence-associated gene expression against Pg. Among the fractions, H4 exhibited the strongest radical-scavenging activity and showed stronger growth inhibition, greater biofilm inhibition, and more pronounced downregulation of several virulence-associated genes than the parent LLH extract under the corresponding experimental conditions. LC–MS/MS analysis tentatively identified α-cyperone as a constituent of H4; however, its individual contribution to these effects was not evaluated. These findings indicate that the fractionation of LLH yielded H4 with enhanced activity across several measured in vitro endpoints, while the specific constituents responsible for these effects remain to be determined. Further studies are required to clarify the individual contributions of the H4 constituents and the mechanisms underlying the observed effects.

## Figures and Tables

**Table 1:** Tentatively identified compounds in *Lycopus lucidus* hexane extract (LLH) based on LC–MS analysis in the positive ion mode.

No	RT (min)	Tentatively Identified Compound	Molecular Formula	Calculated *m/z* [M + H]^+^	Observed *m/z* [M + H]^+^	Mass Error (ppm)
1	7.88	ligustilide	C_12_H_14_O_2_	191.1067	191.1063	–2.09
2	10.56	dihydro-alpha-ionone	C_13_H_22_O	195.1743	195.1739	–2.05
3	12.52	9,12-octadecadiynoic acid	C_18_H_28_O_2_	277.2162	277.2158	–1.44
4	13.60	glycyrrhetinic acid	C_30_H_46_O_4_	471.3469	471.3461	–1.70
5	13.82	13-ketooctadeca-9,11-dienoic acid	C_18_H_30_O_3_	295.2268	295.2273	+1.69
6	13.89	mesterolone	C_20_H_32_O_2_	305.2475	305.2471	–1.31
7	14.52	caryophyllene oxide	C_15_H_24_O	221.1900	221.1903	+1.36
8	14.68	stearidonic acid methyl ester	C_19_H_30_O_2_	291.2319	291.2324	+1.72
9	15.06	echinocystic acid	C_30_H_48_O_4_	473.3625	473.3629	+0.85
10	15.39	6,9,12-octadecatrienoic acid	C_19_H_32_O_2_	293.2475	293.2471	–1.36
11	15.59	13-cis-retinoic acid	C_20_H_28_O_2_	301.2162	301.2155	–2.32
12	15.75	pinolenic acid	C_18_H_30_O_2_	279.2319	279.2312	–2.51
13	16.25	palmitoleic acid	C_16_H_30_O_2_	255.2319	255.2314	–1.96
14	16.33	ursolic acid	C_30_H_48_O_3_	457.3676	457.3681	+1.09
15	16.35	abietic acid	C_20_H_30_O_2_	303.2319	303.2324	+1.65
16	17.46	asclepic acid	C_18_H_34_O_2_	283.2632	283.2625	–2.47

Calculated *m/z* values correspond to protonated molecular ions [M + H]^+^. Mass error (ppm) was calculated as [(observed *m/z* − calculated *m/z*)/calculated *m/z*] × 10^6^. Compounds were tentatively identified based on accurate mass measurements, chromatographic retention behavior, MS/MS fragmentation patterns, and comparison with the NIST spectral library, MassBank, and previously reported spectral data, where available.

**Table 2:** Tentatively identified compounds in the H4 fraction based on LC–MS analysis in positive ion mode.

No	RT (min)	Tentatively Identified Compound	Molecular Formula	Calculated *m/z* [M + H]^+^	Observed *m/z* [M + H]^+^	Mass Error (ppm)
1	8.71	lynestrenol	C_20_H_28_O	285.2213	285.2207	–2.10
2	9.69	valerenic acid	C_15_H_22_O_2_	235.1693	235.1689	–1.70
3	10.46	α-hexylcinnamaldehyde	C_15_H_20_O	217.1587	217.1583	–1.84
4	10.69	monobutyl phthalate	C_12_H_14_O_4_	223.0965	223.0969	+1.79
5	10.70	cinnamic acid	C_9_H_8_O_2_	149.0597	149.0593	–2.68
6	11.5	pinolenic acid	C_18_H_30_O_2_	279.2319	279.2311	–2.87
7	12.66	9,12-octadecadiynoic acid	C_18_H_28_O_2_	277.2162	277.2157	–1.80
8	12.97	α-cyperone	C_15_H_22_O	219.1743	219.1739	–1.83
9	13.33	linolenic acid	C_18_H_30_O_2_	279.2319	279.2325	+2.15
10	16.95	retinol	C_20_H_30_O	287.2369	287.2362	–2.44

Calculated *m/z* values correspond to protonated molecular ions [M + H]^+^. Mass error (ppm) was calculated as [(observed *m/z* − calculated *m/z*)/calculated *m/z*] × 10^6^. Compounds were tentatively identified based on accurate mass measurements, chromatographic retention behavior, MS/MS fragmentation patterns, and comparison with the NIST spectral library, MassBank, and previously reported spectral data, where available.

**Table 3:** DPPH and ABTS radical-scavenging activities and total phenolic and flavonoid contents of *Lycopus lucidus* hexane extract (LLH) and its fractions.

	Radical-Scavenging Activity, IC_50_ (μg/mL) ^1^		Phytochemical Content	
	DPPH	ABTS	TPC (mg CAE/g DM) ^2^	TFC (mg NE/g DM) ^3^
LLH	98.33 ± 2.05 ^c^	89.24 ± 1.67 ^c^	114.48 ± 2.43 ^d^	70.60 ± 3.08 ^a^
H1	78.50 ± 2.68 ^b^	42.05 ± 2.78 ^b^	253.52 ± 0.67 ^a^	51.59 ± 1.73 ^b^
H2	116.57 ± 5.43 ^d^	95.16 ± 2.91 ^d^	118.29 ± 1.17 ^d^	40.80 ± 0.55 ^d^
H3	>500	325.65 ± 4.22 ^g^	89.71 ± 3.09 ^f^	26.10 ± 0.28 ^f^
H4	60.67 ± 3.09 ^a^	28.15 ± 1.21 ^a^	131.14 ± 1.17 ^c^	34.14 ± 1.47 ^e^
H5	>500	>500	81.62 ± 1.78 ^g^	17.27 ± 0.28 ^g^
H6	>500	>500	92.57 ± 1.17 ^f^	24.73 ± 0.73 ^f^
H7	316.67 ± 5.44 ^f^	162.06 ± 1.32 ^f^	145.90 ± 0.67 ^b^	46.29 ± 1.66 ^c^
H8	218.97 ± 2.72 ^e^	118.74 ± 2.46 ^e^	107.33 ± 3.75 ^e^	38.65 ± 1.73 ^d^
Ascorbic acid	8.25 ± 0.89	-	-	-
Trolox	-	5.12 ± 0.42	-	-

Data are presented as mean ± standard deviation (SD) (*n* = 3). Different superscript letters within the same column indicate statistically significant differences according to Duncan’s multiple range test (*p* < 0.05). ^1^ IC_50_, the concentration required to scavenge 50% of DPPH or ABTS radicals; lower IC_50_ values indicate stronger radical-scavenging activity. Ascorbic acid and Trolox were used as positive antioxidant controls for the DPPH and ABTS assays, respectively. ^2^ TPC, total phenolic content, expressed as milligrams of caffeic acid equivalents per gram of dry matter of the extract (mg CAE/g DM). ^3^ TFC, total flavonoid content, expressed as milligrams of naringin equivalent per gram of dry matter of the extract (mg NE/g DM).

**Table 4:** The antimicrobial activity of *Lycopus lucidus* hexane extract (LLH) fractions against *Porphyromonas gingivalis* was determined using disc diffusion, minimum inhibitory concentration (MIC), and minimum bactericidal concentration (MBC) assays.

	Inhibition Zone mg/disc (mm)				Concentrations (μg/mL)	
	0 ^1^	0.5	1	2	MIC	MBC
H1	N.D. ^2^	N.D.	N.D.	11.38 ± 0.03 ^g^	500	500
H2	N.D.	11.95 ± 0.02 ^c^	11.38 ± 0.03 ^g^	20.73 ± 0.09 ^c^	125	250
H3	N.D.	16.18 ± 0.23 ^b^	20.73 ± 0.09 ^c^	21.53 ± 0.09 ^b^	64	250
H4	N.D.	17.47 ± 0.29 ^a^	21.53 ± 0.09 ^b^	23.31 ± 0.16 ^a^	64	250
H5	N.D.	11.02 ± 0.06 ^e^	23.31 ± 0.16 ^a^	13.72 ± 0.22 ^e^	500	500
H6	N.D.	12.16 ± 0.10 ^c^	13.72 ± 0.22 ^e^	12.94 ± 0.06 ^f^	250	≥1000
H7	N.D.	11.56 ± 0.02 ^d^	12.94 ± 0.06 ^f^	12.69 ± 0.12 ^f^	250	≥1000
H8	N.D.	N.D.	12.69 ± 0.12 ^f^	11.26 ± 0.11 ^g^	250	500

Data are presented as mean ± standard deviation (SD) (*n* = 3). Different superscript letters within the same column indicate statistically significant differences according to Duncan’s multiple range test (*p* < 0.05). ^1^ A concentration of 0 μg/mL indicates no LLH fraction treatment. ^2^ N.D., not detected.

## Data Availability

The original contributions presented in this study are included in the article and Supplementary Materials. Further inquiries can be directed to the corresponding author.

## Supplementary Materials

The following supporting information can be downloaded at: https://www.mdpi.com/article/10.3390/antiox15091121/s1, Table S1: Primer sequences used for quantitative reverse transcription PCR (qRT-PCR) analysis.

## Abbreviations

ABTS	2,2′-Azino-bis(3-ethylbenzothiazoline-6-sulfonic acid)
ANOVA	Analysis of variance
DMSO	Dimethyl sulfoxide
DPPH	1,1-Diphenyl-2-picrylhydrazyl
IC_50_	half-maximal inhibitory concentration
KCTC	Korean Collection for Type Cultures
LC-MS/MS	Liquid chromatography–tandem mass spectrometry
LL	*Lycopus lucidus*
LLH	*Lycopus lucidus* hexane extract
MBC	Minimum bactericidal concentration
MIC	Minimum inhibitory concentration
PBS	Phosphate-buffered saline
PDA	Photodiode-array detector
Pg	*Porphyromonas gingivalis*
SD	Standard deviation
TFC	Total flavonoid content
TIC	total ion chromatogram
TPC	Total phenolic content
TSB	Tryptic soy broth
UHPLC	Ultra-high-performance liquid chromatography
