# Supplementary material for: Antioxidant, Antibacterial, and Antivirulence Activities of a Bioactive Fraction from Lycopus lucidus Against Porphyromonas gingivalis
Source: Antioxidants (Basel). 2026 Sep 4;15(9):1121. doi: 10.3390/antiox15091121 (PMC13603838; doi:10.3390/antiox15091121)
Supplement: Supplementary file 1 [file antioxidants-15-01121-s001.zip › antioxidants-4508135-supplementary.pdf]

Table S1. Primer sequences used for quantitative reverse transcription PCR (qRT-PCR) analysis.

**Gene**	Forward primer (5′–3′)	Reverse primer (5′–3′)	GenBank accession number (locus tag)
fimA	CAGCAGGAAGCCATCAAATC	CAGTCAGTTCAGTTGTCAAT	AP009380.1 (PGN_0180)
rgpA	GCCGAGATTGTTCTTGAAGC	AGGAGCAGCAATTGCAAAG	AP009380.1 (PGN_1970)
kgp	AGCTGACAAAGGTGGAGACCAAAGG	TGTGGCATGAGTTTTTCGGAACCGT	AP009380.1 (PGN_1728)
htrA	GAGAGTGGGTATTGGCCGTA	CCCGTTTGGCTGTAGATCAT	AP009380.1 (PGN_0637)
16S rRNA	TGTAGATGACTGATGGTGAAA	ACTGTTAGCAACTACCGATGT	L16492.1

Accession numbers and locus tags correspond to *Porphyromonas gingivalis* ATCC 33277, which is equivalent to KCTC 5352.

.
